# PD-L1 Downregulation and DNA Methylation Inhibition for Molecular Therapy against Cancer Stem Cells in Hepatocellular Carcinoma

**DOI:** 10.3390/ijms241713357

**Published:** 2023-08-29

**Authors:** Caecilia Sukowati, Loraine Kay D. Cabral, Beatrice Anfuso, Francesco Dituri, Roberto Negro, Gianluigi Giannelli, Claudio Tiribelli

**Affiliations:** 1 Liver Cancer Unit, Italian Liver Foundation NPO, AREA Science Park, Basovizza, 34049 Trieste, Italyctliver@fegato.it (C.T.); 2 Eijkman Research Center for Molecular Biology, National Research and Innovation Agency of Indonesia (BRIN), B.J. Habibie Building, Jl. M.H. Thamrin No. 8, Jakarta Pusat 10340, Indonesia; 3 Doctoral School in Molecular Biomedicine, University of Trieste, Piazzale Europa, 1, 34127 Trieste, Italy; 4 Department of Life Sciences, University of Trieste, Piazzale Europa, 1, 34127 Trieste, Italy; 5 National Institute of Gastroenterology, IRCCS Saverio de Bellis Research Hospital, Via Turi 27, Castellana Grotte, 70013 Bari, Italy

**Keywords:** hepatocellular carcinoma, cancer heterogeneity, PD-1/PD-L1, DNA methylation, combination therapy

## Abstract

Hepatocellular carcinoma (HCC) is a heterogeneous cancer characterized by various cellular subtypes. This study investigates the potential of a combination strategy using immunotherapy and epigenetic reprogramming against HCC. We used a transgenic HCC mouse C57BL/6J-TG(ALB1HBV)44BRI/J to assess the dynamics of the programmed death receptor and its ligand (PD-1/PD-L1) and DNA methylation markers. In parallel, PD-L1 RNA silencing was performed in various human HCC cell lines, while combination therapy was performed in a co-culture system using long-term exposure of 5-Azacytidine (5-AZA) and an anti-PD-L1. Data from the mouse model showed that the expressions of *Pdcd1, Pdcd1l1*, and DNA methyltransferase 1 (*Dnmt1*) were significantly higher in HCC as compared to the wild-type mice (*p* < 0.01), supported by the high presence of PD-L1 methylated DNA. In HCC cell lines, *PD-L1* silencing was accompanied by *DNMT1* reduction, mostly noted in aggressive HCC cell lines, followed by the dysregulation of the cancer stem cell marker EpCAM. In combination therapy, the growth of HCC cells and lymphocytes was limited by the PD-L1 antibody, further reduced in the presence of 5-AZA by up to 20% (*p* < 0.001). The data demonstrated that combination therapy might be an option as a potential treatment for heterogeneous HCC.

## 1. Introduction

Global epidemiology data for cancer predicts liver cancer to be the sixth most commonly diagnosed cancer and the fourth leading cause of cancer-related mortality worldwide [1]. Hepatocellular carcinoma (HCC) accounts for about 90% of liver cancer cases, with cirrhosis as the strongest underlying condition [2]. HCC is highly heterogeneous, within a tumor and among individuals, thus affecting disease progression, classification, prognosis, and, naturally, cancer cell resistance and response to molecular therapy [3].

Immune checkpoint inhibitors (ICIs) against the programmed death receptor 1 (PD-1) protein and its ligand PD-L1 (B7-H1, CD274) also have been a focus in cancer immunology and oncology. The Food and Drug Administration (FDA) had approved ICIs inhibitors for the treatment of many cancer types including HCC, comprising nivolumab (antibody against PD-1), and a combination of atezolizumab (antibody against PD-L1) and bevacizumab (anti-vascular endothelial growth factor/anti-VEGF) [4,5]. Nevertheless, despite the success of ICIs in treating various cancers, a large proportion of HCC patients still showed low response rate due to primary or acquired resistance mechanisms [6], which are highly correlated with individual cellular heterogeneity and diverse immune systems.

Apart from gene and protein expressions, epigenetic plays a significant role in carcinogenesis. DNA methylation, a biological process by which methyl groups are added to the DNA molecule by DNA methyltransferases (DNMTs), has become a promising interest. DNA methylation regulates the expression of genes involved in cellular proliferation, immune checkpoints, and the response to anti-cancer drugs [7]. In HCC, enormous differential profiles of DNA methylation had been demonstrated in both clinical samples and preclinical models, correlated with clinical manifestations [8]. A so-called epigenetic reconditioning using a DNA demethylation agent was able to induce HCC cell differentiation by increasing the expression of mature hepatocyte markers from liver progenitor cancer cells [9] and inducing a tumor suppressor gene [8]. However, until now, only a few studies indicated the potential of combining epigenetic drugs with PD-1/PD-L1 targeting for cancer treatment [10,11], even fewer in a selective cell population.

The usability of PD-L1 to represent a predicting biomarker for HCC aggressiveness and patient survival is also a characteristic shared with hepatic cancer stem cell (CSC) markers. Until now, very limited data were available on CSC sensitivity to anti-PD-L1/PD-1 and other immunotherapy strategies [12]. Thus, it is reasonable to investigate the contribution of hierarchical heterogeneity, in particular involving the treatment-resistant CSC, to cellular sensibility to treatment.

Here, we aimed to investigate the synergy between epigenetic reprogramming using DNA demethylation and PD-1/PD-L1 immunotherapy as a potent and novel cancer treatment and whether PD-L1 might be related to a certain CSC profile.

## 2. Results

### 2.1. Dynamics of PD-1/PD-L1 during Hepatocarcinogenesis

For in vivo animal study, male HBV-transgenic mice C57BL/6J-TG(ALB1HBV)44BRI/J (TG) [13], together with their wild-type counterpart C57BL76J (WT), were used as animal models. Liver tissue samples were collected from different stages of liver damage in TG mice (age 3 months: inflammation, 6 months: early hepatic injury, 9 months: pre-neoplastic lesion, and 12 months of age: neoplasia).

**Figure 1:**
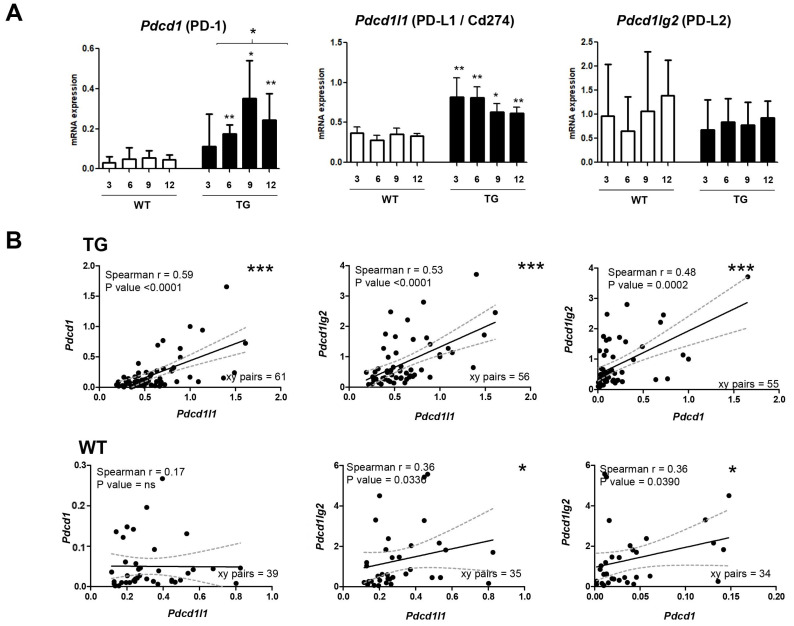
The expression of PD-1/PD-L1/2 axis in in vivo transgenic mouse model. (**A**) The mRNA expression of *Pdcd1* (PD-1), *Pdcd1l1* (PD-L1), and *Pdcd1lg2* (PD-L2) following hepatocarcinogenesis in TG mice, compared to their WT counterparts. Statistical test: ANOVA between groups; *t*-test of TG to WT in same age group; * *p* < 0.05, ** *p* < 0.01. (**B**) Analysis of linear correlations of the mRNA expressions of *Pdcd1*, *Pdcd1l1*, and *Pdcd1lg2* in both TG and WT mice. Statistical test: * *p* < 0.05, ** *p* < 0.01, *** *p* < 0.001. TG: transgenic HCC mouse, WT: wild-type mouse.

Using 112 hepatic tissue samples of TG (*n* = 69) and their control WT mice (*n* = 43), the activations of both PD-1 and PD-L1 in TG mice were observed using reverse transcription–quantitative real time PCR (RT–qPCR). The expression of the *Pdcd1* gene (encoding PD-1) was increased alongside the progression of the liver disease, from inflammation to the development of the tumor (ANOVA *p* < 0.05). In parallel, the expression of the *Pdcd1l1* gene (encoding PD-L1) was significantly amplified already in early liver injury. *Pdcd1l1* expression in TG was significantly upregulated compared to that in WT mice (*p* < 0.05) at each age-point. The highest expressions of both *Pdcd1* and *Pdcd1l1* were noticeable in pre-tumoral stage nodules in 9-month-old mice. Conversely, the expression of *Pdcd1lg2* (encoding PD-L2) did not show any significant differences in term of hepatocarcinogenesis (Figure 1A).

The correlations among the mRNA expressions of *Pdcd1*, *Pdcd1l1*, and *Pdcd1lg2* in mouse models, both TG and WT, were analyzed. As expected, from 62 paired data of TG samples, the correlation between *Pdcd1* and *Pdcd1l1* showed the highest score (Spearman correlation R > 0.6, *p* < 0.0001) compared to other pairs. Correlations between *Pdcd1* and *Pdcd1lg2* (*p* < 0.0001), and between *Pdcd1l1* and *Pdcd1lg2* (*p* = 0.0002), were also found. On the other hand, in 43 paired data of WT samples, these correlations were not observed, except for rather weak correlations with *Pdcd1lg2* (Spearman correlation R = 0.36, *p* = 0.03) (Figure 1B).

### 2.2. Relevance of DNA Methylation during Hepatocarcinogenesis

**Figure 2:**
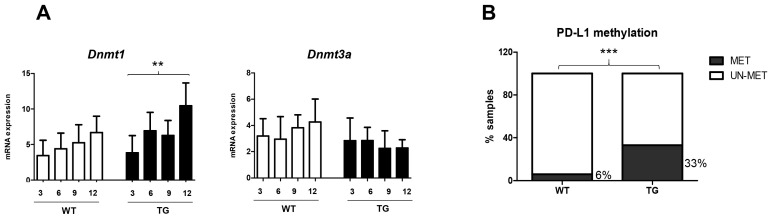
The relevance of DNA methylation in transgenic HCC mouse model. (**A**) Gene expression of DNMTs following hepatocarcinogenesis. (**B**) The proportion of PD-L1 DNA methylation in TG vs. WT mice. Percentage represents the proportion of methylated samples. Statistical test: ** *p* < 0.01, *** *p* < 0.001. DNMTs: DNA methyltransferases, TG: transgenic HCC mouse, WT: wild-type mouse, MET: methylated, UN-MET: unmethylated.

As for the PD-1/PD-L1 axis, the dynamics of DNMTs were analyzed in the mouse model. Using a similar set of mice samples as above, the mRNA expressions of *Dnmt1* and *Dnmt3a* were also assessed by RT-qPCR. As shown in Figure 2A, the increase of *Dnmt1* was significant in TG (ANOVA *p* < 0.05), to a higher extent compared to WT mice. There was no significant change for *Dnmt3a* either for TG or WT mice.

We then checked the DNA methylation status of the PD-L1 in 34 samples (18 TG and 16 WT). By using an in-house method of methylation-specific PCR (MS-PCR) to detect DNA methylation in older animals (9–12 months), we showed that PD-L1 DNA methylation was notably present in TG compared to WT (6/18 vs. 1/16; 33% vs. 6%, for TG and WT animals, respectively; *p* < 0.001) (Figure 2B).

### 2.3. Silencing of PD-L1 mRNA in Human HCC Cell Lines

**Figure 3:**
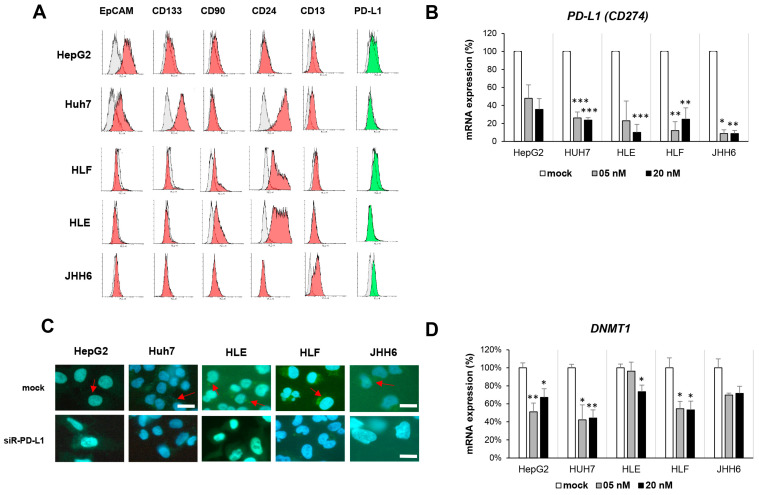
The PD-L1 in HCC cell lines HepG2, Huh7, HLE, HLF, and JHH-6. (**A**) Phenotype of HCC cell based on CSC markers and PD-L1 protein. Grey color indicates unstained control, red indicates the positivity of CSC markers, and green indicates the positivity of PD-L1. (**B**) Downregulation of *PD-L1* mRNA in cells following 48 h silencing using siRNA against human *PD-L1*. (**C**) Immunofluorescence staining of PD-L1 protein following siR-PD-L1 of 20 nM. Bar = 50 µm; red arrow indicates PD-L1 protein. (**D**) Dysregulation of *DNMT1* mRNA in cells following 48 h silencing using siRNA against human *PD-L1*. Statistical test: *t*-test; * *p* < 0.05, ** *p* < 0.01, *** *p* < 0.001 vs. mock.

Continuing data in vivo, to observe whether the gene modulation of PD-L1 differently affects HCC cells, we used different HCC cell lines, representing HCC cellular heterogeneity. Figure 3A shows the different phenotype of each cell line based on CSC markers and PD-L1 expressions by flow cytometry. In line with previous studies, EpCAM was the CSC apparent in progenitor subtypes HepG2 and Huh7 [8]. The expression of PD-L1, however, varies among cell lines with the highest percentage noticed in JHH-6.

The PD-L1 (*CD274*) gene was silenced by small interference RNA (siRNA) against *CD274*. Following gradual siRNA concentration up to 20 nM, *PD-L1* mRNA expression in all cell lines was significantly downregulated, up to 90% compared to its mock control. The siRNA concentration of 20 nM was able to reduce the *PD-L1* mRNA from 100% (mock) to around 50 ± 28% for HepG2, 24 ± 3% for Huh7, 30 ± 21% for HLE, 18 ± 13% for HLF, and 9 ± 4% for JHH-6, represented as mean ± sd. The mRNA downregulation was then validated with PD-L1 protein reduction in all cell lines, showed by immunofluorescence (IF) staining (Figure 3B,C).

Interestingly, in line with data in the animal model, the downregulation of *PD-L1* in HCC cells was accompanied by the decrease of *DNMT1* mRNA, both for a concentration of 5 and 20 nM, until up to a 60% decrease in Huh7 cells (*p* < 0.01) (Figure 3D). It indicates a possible correlation between PD-L1 and DNA methylation.

### 2.4. Combination Therapy Inhibits HCC Growth

**Figure 4:**
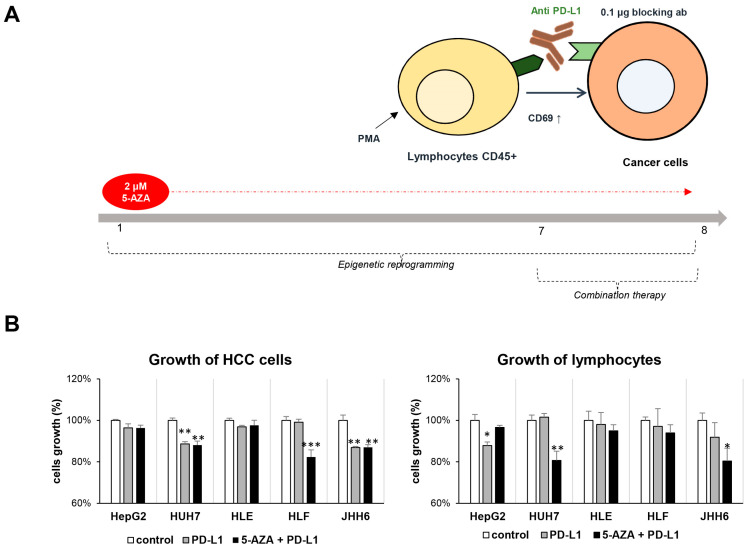
Co-culture system for combined therapy using anti-PD-L1 and DNA methylation inhibitor 5-AZA. (**A**) The representative protocol of co-culture between HCC cells and immortalized lymphocytes in the presence of nontoxic concentration of 5-AZA and a functional antibody against PD-L1. (**B**) The growth of HCC cells and lymphocytes following combined therapy. Statistical test: *t*-test; * *p* < 0.05, ** *p* < 0.01, *** *p* < 0.001 vs. control.

Taking the data above of the potential of PD-L1 and DNA methylation, a combination therapy using a functional antibody against human PD-L1 and a nontoxic concentration of 5-AZA, a DNA methylation inhibitor, was conducted in a long-term experimental setting. As shown through the graphical method in Figure 4A, HCC cells were adapted with the nontoxic concentration of 2 µM of 5-AZA for 7 days, then plated together in a cell-to-cell contact with phorbol myristyl acetate (PMA)-activated Jurkat cells for 24 h in the presence of 0.1 µg of functional antibody against PD-L1. Following PMA treatment, the Jurkat cells increased their expression of CD69 protein, a classical early marker of lymphocyte activation after stimulation.

Viability tests showed that in line with HCC cellular heterogeneity and the basal expression of PD-L1, the growth of the HCC cells was altered in the presence of Jurkat cells, anti-PD-L1, and 5-AZA. The blocking of PD-L1 by using the antibody reduced (10–20%) the growth of three cell lines with higher PD-L1 expressions (JHH-6, Huh7, and HLF) compared to the control alone (HCC cells + Jurkat, *p* < 0.05). However, only in one out of five cell lines (HLF) was the effect of the combination between 5-AZA and anti-PD-L1 significant compared to anti-PD-L1 alone. When cell growth was assessed in Jurkat cells, the growth of the Jurkat cells was slightly lower in the presence of anti-PD-L1, especially noticed for HepG2 (*p* < 0.05) and JHH-6. Interestingly, the co-culture between Jurkat cells and 5-AZA-treated cells significantly reduced the growth of Jurkat cells in culture together with Huh7 and JHH-6 (*p* < 0.05) (Figure 4B).

### 2.5. The Expression of PD-L1 Is Related to That of CSC Marker EpCAM

**Figure 5:**
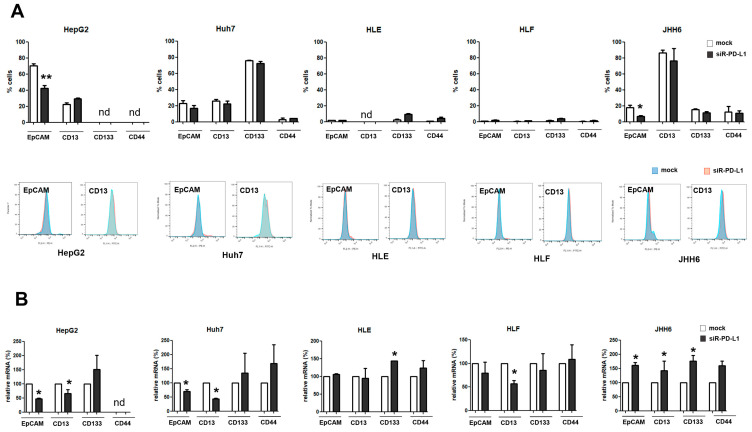
The *PD-L1* mRNA affected the expression of hepatic CSC markers. (**A**) Flow cytometric analysis of CSC markers EpCAM, CD13, CD133, and CD44 following PD-L1 silencing in HepG2, Huh7, HLE, HLF, and JHH-6 cells. Above: number of cells; below: representative histogram plot. Blue color indicates mock, red indicates the PD-L1-silenced samples. (**B**) Gene expression analysis of above markers. Data were taken from at least 10,000 events for flow cytometry and from 3 independent experiments for both analysis. Data is presented as mean ± sd. Statistical test: *t*-test; * *p* < 0.05, ** *p* < 0.01 vs. mock; nd: not detectable.

Upon PD-L1 silencing, the expression and the presence of several CSC markers were analyzed by RT-qPCR and flow cytometry. As shown in Figure 5A, flow cytometric analysis showed a substantial reduction of the EpCAM^+^ cells in HCC cell lines expressing this marker, the HepG2, Huh7, and JHH-6 (*p* < 0.05). Except for that in JHH-6, EpCAM downregulation was also noticed in mRNA analysis (Figure 5B). As for *CD13/ANPEP* mRNA, a similar trend was noticed for all HCC cells (*p* < 0.05) where PD-L1 silencing downregulated *CD13* expression, with the exception of JHH-6. Different was the result for CD133/Prom-1 where the increase in the number of CD133^+^ cells and/or the upregulation of *CD133* mRNA expression was observed in most of the cell lines analyzed, indicating that CD133 might be negatively correlated with PD-L1.

Data in vitro were then compared with data in vivo in TG mice. We previously reported the upregulation of various CSC markers in this mouse model, including the drastic upregulation of *Cd133* and *Epcam* during hepatocarcinogenesis [14]. Analyzing paired gene expression data from up to 62 TG samples showed that the mRNA expression of *Pdcd1l1* was linearly associated with *Epcam* (Spearman correlation R = 0.40, *p* = 0.0101) only in TG animals. No association was noticed for either *Cd133* or *Thy1*. As for WT animals, analyzing paired data from up to 43 samples, no associations were observed for all three genes analyzed, except between *Pdcd1* and *Thy*-1 (Table 1).

## 3. Discussion

Recent breakthroughs in immunotherapies using ICIs, including those targeting PD-1 and its ligand PD-L1, represent a promising option for HCC treatment. In the liver, PD-1 is mostly noticed in tumor-infiltrating CD8+ T cells, while PD-L1 is stained positive in tumor cells and also tumor-infiltrating lymphocytes [15]. Hepatic membrane-bound PD-L1 expression represents a predicting biomarker for HCC aggressiveness and patient survival [16,17,18]. PD-1 has two ligands, PD-L1 (B7-H1) and PD-L2 (CD273, B7-DC) [19]. Even though PD-L2 has a higher affinity with PD-1 compared to PD-L1, PD-L2 is only expressed in antigen-presenting cells. In contrast, PD-L1 is expressed in cancer cells where PD-L1 upregulation was also reported in various cancer types. It was positively correlated with disease stages and poor prognosis [20].

In this study, we showed in an animal model that the expressions of both *Pdcd1/Pdcd1l1* (PD-1/PD-L1) and *Dnmt1* were higher in TG HCC mice than those in the control normal animals (WT mice). As expected, the expression of *Pdcd1lg2* (PD-L2) between TG and WT was comparable. Using an in-house MS-PCR technique, the percentage of methylated PD-L1 DNA was also significantly high in HBV-TG animals.

The trend of *Pdcd1* and *Dnmt1* gene upregulations was increasing along with the progression of liver damage from 3 to 12 months of age, representing the natural history of hepatocarcinogenesis from early hepatic damage, inflammation, dysplasia, and finally neoplasia. These data were in line with previous observations that this model expressed genes involved in the regulation of immunological response in early events that progressed to more advanced damage, such as fibrosis, in later events [14,21]. It is also important to notice that a consistently slight increase of *Dnmt1* in WT mice was noticed with aging. It was previously demonstrated that *Dnmt1* loss in postnatal hepatocytes caused global hypomethylation and enhanced DNA damage response [22].

Regarding *Pdcd1l1*, a significant two-fold increase was noticed in HBV-TG mice, already started at 3 months of age. This upregulation was maintained during the progression of hepatic damage. Interestingly, a strong linear correlation between *Pdcd1* and *Pdcd1l* was observed indicating that either PD-1 or the PD-L1 molecule can be used to target this checkpoint axis. It was previously demonstrated that the inhibition of PD-L1 resulted in the reactivation of the exhausted immune cells in the tumor microenvironment, leading to the elimination of cancer cells [23]. Since PD-L1 is the molecule expressed in markedly heterogeneous cancer cells, we then focused our attention on PD-L1.

The relevance of PD-L1 in heterogeneous cells and its relationship with DNA methylation inhibition was assessed in vitro. HCC cells JHH-6, HLE, and HLF were classified in the S1/TGFβ-Wnt-activated subtype, while cells HepG2 and Huh7 were in the S2/progenitor subtype [24]. We showed that PD-L1 was also expressed in various amounts in these cells and that silencing of the *PD-L1* gene significantly reduced not only mRNA but also the PD-L1 protein. Interestingly, the downregulation of *PD-L1* was also accompanied by the downregulation of *DNMT1*.

This study expanded previous studies on various cancers. It was demonstrated in non-small small cell lung carcinoma that PD-L1 expression was regulated by both DNA methylation and Nuclear Factor Kappa B (NF-kB) during epithelial-mesenchymal transition (EMT) signaling [25]. In an HCC study, highly upregulated DNMT1 was positively correlated with PD-L1 overexpression in sorafenib-resistant cells. PD-L1 depletion by shRNA in HepG2 and Huh7 decreased protein and mRNA levels in both PD-L1 and DNMT1. Further analysis showed that PD-L1 was shown to regulate DNMT1 through the Signal Transducer and Activator of Transcription 3 (STAT3) signaling pathway. Knockdown of PD-L1 induced DNMT1-dependent DNA hypomethylation and restored the expression of methylation-silenced cadherin 1 (CDH1), a metastasis suppressor [26]. In a triple-negative breast cancer of The Cancer Genome Atlas Program (TCGA) cohort, the methylation statuses of PD-1 and PD-L1 were significantly correlated with mRNA levels indicating a strong epigenetic regulation of transcriptional activity. DNA methylation status was strongly associated with a distinct immune cell infiltration pattern [27].

We then investigated whether a combination therapy between PD-L1 blocking and DNA methylation inhibition would be beneficial. We used the co-culture between immortalized lymphocyte Jurkat cells. which were previously activated with PMA [28], and induced the expression of CD69. Before the co-culturing, HCC cells were primed with a low concentration of 5-AZA of 2 µM, a concentration lower than the lethal concentration 50 (LC_50_) in all cell lines analyzed. In addition, 5-AZA in a nontoxic concentration was able to induce tumor suppressor gene *SOCS1* [8]. We showed that a combination therapy using 5-AZA and an antibody against PD-L1 reduced the growth of both lymphocytes and HCC cell lines, with a greater extent observed for the lymphocytes (4 out of 5 co-culture pairs). As expected, this beneficial effect was noticeable in a co-culture system with HCC cells with high PD-L1 expression.

Our data on this epigenetic and immunotherapy are in accordance with other studies in other cancers. In esophageal cancer, the combination of epigenetic modulation by 5-AZA-2′-deoxycytidine and PD-1/PD-L1 blocking was reported to be a potential T cell-based immunotherapy. The blocking of PD-1/PD-L1 increased the specific CTL-induced lysis of HLA-A2+/MAGE-A11+ tumor cell lines treated with 5-AZA-2′-deoxycytidine [11]. In oral squamous cell carcinoma, the expression levels of DNMT1 were also correlated with the immunosuppressive molecules and tumor-promoters such as PD-L1, indicating a worse prognosis. The inhibition of DNMT1 improved tumor microenvironment and delayed tumor growth [29].

In pancreatic ductal adenocarcinoma, it was shown that immunotherapy using anti-PD-L1 can be potentiated with epigenetic therapy (DZNep and 5-AZA) by increasing cancer-associated antigen expression and increasing T cell trafficking across the immunosuppressive tumor microenvironment [30]. It is also in line with a previous study in HCC cells (HepG2, Hep3B, and Hepa1-6) treated with 5-AZA (and DZNep) with and without anti-PD-L1. This combination induced the significant upregulation of Th1 chemokines in the cells. In in vivo C57/LJ immunocompetent mouse models, combination treatment also significantly reduced tumor growth [31].

Another important finding of our study is the association between PD-L1 and the CSC marker EpCAM, a determinant marker for progenitor HCC subtypes HepG2 and Huh7. EpCAM is one of the most common CSC markers, not only in HCC but also in other cancers. HCCs expressing EpCAM were aggressive, and its high expression was associated with unfavorable prognostic factors [32,33]. Our data supports previous data on breast cancer showing that the expression of PD-L1 was strongly associated with stem cell-like cells where the CSC population (EpCAM^+^CD44^hi^CD24^lo^) had a higher level of PD-L1 compared to their counterparts (EpCAM^lo/neg^CD44^lo^CD24^hi^) [34,35].

A previous study on HCC, however, showed that even though PD-L1 was frequently expressed in stem cell features of HCC, the presence of PD-L1 was positively associated with cytokeratin 19 (CK-19) and Sal-like protein 4 (SALL-4), but not with EpCAM [36]. We assume that this discrepancy is due to the heterogeneity of the HCC samples. The mentioned study used immunohistochemistry (IHC) staining in clinical specimens and it was coherent with our previous data on the distribution of CSC markers in clinical samples, both from Western and Eastern cohorts. This study showed that *EpCAM* was one of the markers with the highest variability in terms of gene expression [37].

Following the data in vitro, we looked again at the data in vivo on the dysregulation of CSC markers during hepatocarcinogenesis in an animal model. The expression of *Epcam* in TG animals was drastically increased during the progression of liver damage [14]. We found that, also in this model, *Pdcd1l1* was positively correlated with the expression of *Epcam* only in the TG animals, while it was not associated with *Cd133* and *Thy-1*. Once again, it emphasizes the importance of PD-L1 targeting in the aggressive CSC cells with the EpCAM phenotype.

Recently, we had presented parallel data of in silico and in vitro analysis on various proto-oncogenes as candidate targets for HCC treatments. We showed that PD-L1 silencing was effective at downregulating almost all proto-oncogenes, especially noticed in HepG2 and Huh7, cells with a high expression of EpCAM^+^ cells [38]. This demonstrates an effective advantage of PD-L1 targeting, better to an extent in a combination therapy with epigenetic targeting using DNA methylation inhibitor, for at least in the progenitor HCC subtype among heterogeneous HCC cells.

To summarize, this study supports that the targeting of the PD-1/PD-L1 checkpoints, in particular PD-L1, is a promising approach for HCC, including the reduction of the CSC EpCAM. The data also demonstrate that the combination of immunotherapy and epigenetic therapy might be an option as a potential treatment. However, understanding cellular heterogeneity is essential to define its efficacy.

## 4. Materials and Methods

### 4.1. Transgenic Mouse Model

Male HBV-transgenic mice C57BL/6J-TG(ALB1HBV)44BRI/J (TG/HCC) [13], together with their wild-type counterparts (C57BL76J) (WT), were used as animal models. All animals were maintained at the animal facility of the University of Trieste. The experiment was carried out in accordance with the Guide for the Care and Use of Laboratory Animals. The protocol and animal study were approved by the ethical committee of the University of Trieste and by the responsible administration of the Ministry of Health of the Republic of Italy (D 699/2020-PR).

### 4.2. In Vitro Models

Several human HCC cell lines representing in vitro heterogeneous HCC cells were used as in vitro models. HCC cell lines HLE, HLF, and JHH-6 are categorized into the S1/TGFβ-Wnt-activated subtype, while HepG2 and Huh7 are placed into the S2/progenitor subtype [24]. Immortalized lymphocyte Jurkat cells were grown in RPMI-1640 medium (Invitrogen, Waltham, MA, USA) supplemented with 10% fetal bovine serum, containing penicillin (100 U/mL), streptomycin (100 mg/mL), and glutamine (2 mM). Cell culture media and the expansion protocol of HCC cells were reported previously [8]. Expansion was performed in 80% confluence by 0.05% trypsin detachment for HCC cells and replating with lower density for Jurkat cells.

### 4.3. Reverse Transcription–Quantitative Real Time PCR (RT–qPCR)

Total RNA from mouse liver tissues and human cell lines were purified using TriReagent (Sigma-Aldrich, Burlington, MA, USA). Reverse transcription (RT) was performed to obtain cDNA from 1 µg of purified RNA with the High Capacity cDNA Reverse Transcription Kits (Applied Biosystem, Waltham, MA, USA) according to the manufacturer’s protocol. PCR amplification was carried out in a 15 µL reaction volume containing 25 ng cDNA, PowerUp SYBR Green Master Mix (Life Technologies, Carlsbad, CA, USA), and 250 nM of gene-specific forward and reverse primers. The reaction was run in a CFX 9600 real-time PCR system (Bio-Rad, Hercules, CA, USA). The primer sequences for the detection of the desired gene are listed in the Supplementary Material (Table S1). The primer sequences from human and mouse CSCs and progenitor cell markers, together with their respective reference housekeeping genes, are listed in our previous works [14,37].

### 4.4. DNA Isolation and Methylation-Specific PCR (MS-PCR)

The methylation status of PD-L1 in mouse tissues were defined by methylation-specific PCR (MS-PCR). MethPrimer 2.0 Primer Design© web tool [39] was employed to design the MS-PCR primer for mouse PD-L1 methylation in this study. Methylated primer pairs were designed using the mRNA sequence of Mus musculus CD274 antigen (Cd274) (NM_021893.3). Primers (5′-CGAGTATAGTCGAATTTTCGGT-3′; 5′-AAATAAATAAAATATATTACCCAACCCG-3′) flank the region between 3361 to 3494 base pairs (bp). This resulted to a PCR product of 134 bp.

Mouse genomic DNA (gDNA) was extracted from frozen tissues, and a total of 1 ug of DNA eluent was subjected for bisulfite conversion using an EZ DNA Methylation-Direct kit (Zymo Research, Irvine, CA, USA), based on the manufacturer’s protocol. MS-PCR reaction was carried out in a 15 µL PCR reaction volume containing about 200 ng bcDNA, 1X Power-Up SYBR Master Mix (Thermo Scientific, Waltham, MA, USA), and 250 nM of methylation-specific forward and reverse primers. The presence of a methylated-PD-L1 sample was indicated by the detection of the melting peak temperature of PD-L1 methylated primers of 71.0 °C.

### 4.5. PD-L1 RNA Silencing

*CD274 (PD-L1)* RNA silencing was performed by using Hs siRNA against CD274 (Thermo Fisher). Scrambled siRNA was used as a mock control in each experiment. Briefly, cells were seeded in a 6-well plate until reaching 80% confluence in optimal conditions. The silencing protocol was based on the manufacturer’s suggestion for the transfection agent (SilentFect, BioRad). The transfection mixture was conjugated for 45 min before being added to cells in a glutamine-free medium followed by incubation for 8 h. A complete medium was then added for the silencing treatment for a total of 48 h.

### 4.6. Flow Cytometry

Following cell identification and RNA silencing, cells were detached, washed, and incubated with specific first antibodies for 60 min on ice in the dark. The first antibodies used were anti-CD274/PD-L1 (Thermo Fisher), anti-CD13/ANPEP (WM15, Santa Cruz Biotechnology, Dallas, TX, USA), anti-EpCAM (VU-1D9, Santa Cruz Biotechnology), anti-CD133/Prom-1 (AC133, Miltenyi Biotec, Bergisch Gladbach, NR, USA), anti-CD24 (32D12, Miltenyi Biotec), and anti-CD44 (F10-44-2, Abcam, Cambridge, UK). After two washings with PBS containing 0.5% bovine serum albumin (BSA), when necessary, the cells were then incubated with fluorescence-conjugated secondary antibody for another 60 min on ice in the dark. Flow cytometric analysis was performed immediately in a flow cytometer (FACS Accuri, BD Biosciences, San Jose, CA, USA). Ten thousand events were analyzed per sample. Data were presented as a mean ± sd for at least three independent experiments.

### 4.7. Immunofluorescence

Immunofluorescence (IF) analysis was performed on PD-L1 silenced cells. In brief, cells were plated in coverslip before proceeding to RNA silencing. Upon 48 h, cells were washed twice with PBS and fixed with 4% paraformaldehyde for 15 min. First antibody conjugation using anti PD-L1 was performed at 4 °C overnight in a humid chamber. Secondary conjugation was then performed using FITC-conjugated IgG anti-mouse for 1 h at room temperature. Hoechst 33328 was used to stain the nucleus. Protein positivity of IF was observed by using a fluorescence microscope Leica DM2000 (Leica Camera AG, Solms, Germany).

### 4.8. Co-Treatment of Anti PD-L1 and DNMT1 Inhibitor

5-Azacytidine (5-AZA, Sigma Aldrich, St Louis, MO, USA), a DNMT inhibitor, was used for epigenetic therapy as we previously reported [8]. HCC cells were plated with a concentration of 25,000 cells/cm2 (12,500 cells/mL for JHH-6) for 24 h then exposed to 2 μM of 5-AZA for 7 days. In parallel, Jurkat cells were activated using 100 ng/mL of phorbol myristyl acetate (PMA, Sigma Aldrich) 24 h. Co-culture between HCC cells and activated Jurkat was performed in the addition of 0.1 μg functional antibody against PD-L1 (MIH1, Thermo Fisher). Cell growth, both for HCC cells and lymphocytes, was determined by 3(4,5-dimethyl thiazolyl-2)-2,5 diphenyltetrazolium assay (MTT, Sigma Aldrich) after 24 h.

### 4.9. Statistical Analysis

Statistical significance was calculated using the software GraphPad Prism version 8.0 (GraphPad Software, Inc., La Jolla, CA, USA). For mouse tissue samples, continuous variables of mRNA expression were calculated following a normality test, using ANOVA for differences within the group, *t*-test for comparison of HBV-TG to WT in the same age group, and Spearman correlation R for linear analysis. Data in vitro were obtained from at least three independent experiments and are expressed as mean ± SD. All tests were two-tailed, and statistical significance was set to *p*-value < 0.05 and reported as * *p* < 0.05, ** *p* < 0.01, and *** *p* < 0.001.

## Figures and Tables

**Table 1:** Spearman linear correlations between mRNA expressions of PD-1/PD-L1 axis and cancer stem cell markers in mouse model.

	TG			WT		
	PD-1	PD-L1	PD-L2	PD-1	PD-L1	PD-L2
Thy-1	XY pairs: 39	XY pairs: 42	XY pairs: 34	XY pairs: 35	XY pairs: 39	XY pairs: 31
	R: 0.15	R: 0.26	R: 0.04	R: 0.69	R: 0.31	R: 0.21
	*p*: ns	*p*: ns	*p*: ns	*p* < 0.0001	*p*: 0.0546	*p*: ns
Prom-1	XY pairs: 60	XY pairs: 62	XY pairs: 54	XY pairs: 39	XY pairs: 43	XY pairs: 35
	R: −0.11	R: 0.04	R: −0.10	R: −0.01	R: −0.00	R: −0.05
	*p*: ns	*p*: ns	*p*: ns	*p*: ns	*p*: ns	*p*: ns
Epcam	XY pairs: 40	XY pairs: 41	XY pairs: 38	XY pairs: 26	XY pairs: 28	XY pairs: 25
	R: 0.59	R: 0.40	R: 0.30	R: 0.20	R: −0.21	R: −0.22
	*p*: 0.0010	*p*: 0.0101	*p*: 0.0633	*p*: ns	*p*: ns	*p*: ns

Analysis of linear correlations of the mRNA expressions of Pdcd1l1 and CSC markers *Epcam*, *Prom1*, and *Thy-1* in both TG and WT mice. Statistical test: Spearman correlation R; TG: transgenic HCC mouse; WT: wild-type mouse.

## Data Availability

The data that support the findings of this study are available on request from the corresponding author.

## Supplementary Materials

The supporting information can be downloaded at https://www.mdpi.com/article/10.3390/ijms241713357/s1. References [40,41,42,43,44] are cited in Supplementary Materials.
