# Supplementary material for: PD-L1 Downregulation and DNA Methylation Inhibition for Molecular Therapy against Cancer Stem Cells in Hepatocellular Carcinoma
Source: Int J Mol Sci. 2023 Aug 29;24(17):13357. doi: 10.3390/ijms241713357 (PMC10487900; doi:10.3390/ijms241713357)
Supplement: Supplementary file 1 [file ijms-24-13357-s001.zip › ijms-2494031-supplementary.pdf]

SUPPLEMENTARY MATERIALS

Article

PD-L1 Downregulation and DNA Methylation Inhibition for Molecular Therapy against Cancer Stem Cells in Hepatocellular Carcinoma

Caecilia Sukowati [1,2,*], Loraine Kay D. Cabral [1,3], Beatrice Anfuso [4], Francesco Dituri [5], Roberto Negro [5], Gianluigi Giannelli [5] and Claudio Tiribelli [1]

[1] Liver Cancer Unit, Italian Liver Foundation NPO, AREA Science Park, Basovizza, 34049 Trieste, Italy; ctliver@fegato.it (C.T.)
[2] Eijkman Research Center for Molecular Biology, National Research and Innovation Agency of Indonesia (BRIN), B.J. Habibie Building, Jl. M.H. Thamrin No. 8, Jakarta Pusat 10340, Indonesia
[3] Doctoral School in Molecular Biomedicine, University of Trieste, Piazzale Europa, 1, 34127 Trieste, Italy
[4] Department of Life Sciences, University of Trieste, Piazzale Europa, 1, 34127 Trieste, Italy
[5] National Institute of Gastroenterology, IRCCS Saverio de Bellis Research Hospital, Via Turi 27, 70013 Castellana Grotte, Bari, Italy
* Correspondence: caecilia.sukowati@fegato.it; Tel.: +39-040-375-7926

Table S1. Primer used in this study

Gene	Forward (5' → 3')	Reverse 5' → 3'	Ref.
Homo sapiens			
PDCD1/PD-1	ATGGTTCTTAGACTCCCCAG	CTCCGATGTGTTGGAGAAGC	[1]
CD274/PD-L1	AAAGTCAATGCCCCATACAA	ACATGTCAGTTCATGTTCAGAG	[1]
PD-L2	GTCTTGGGAGCCAGGGTGAC	TGAAAAGTGCAAATGGCAAGC	[2]
DNMT1	CCATCAGGCATTCTACCA	CGTTCTCCTTGTCTTCTCT	[3]
DNMT3a	TATTGATGAGCGCACAAGAGAGC	GGGTGTTCCAGGGTAACATTGAG	[3]
Mus musculus			
Pdcd1	ACCCTGGTCATTCACTTGGG	CATTTGCTCCCTCTGACACTG	[4]
Cd274	GCTCCAAAGGACTTGTACGTG	TGATCTGAAGGGCAGCATTTC	[4]

Pdcd1lg2	CTGCCGATACTGAACCTGAGC	GCGGTCAAAATCGCACTCC	[4]
Dnmt1	CCTAGTTCCGTGGCTACGAGGAGAA	TCTCTCTCCTCTGCAGCCGACTCA	[5]
Dnmt3	ATGTGGTTCGGAGATGGCAAG	AGATGGCTTTGCGGTACATGG	[5]

References

1. Uhercik M, Sanders AJ, Owen S, Davies EL, Sharma AK, Jiang WG, et al. Clinical Significance of PD1 and PDL1 in Human Breast Cancer. Anticancer Res. 2017;37:4249–54.

2. Hassan SS, Akram M, King EC, Dockrell HM, Cliff JM. PD-1, PD-L1 and PD-L2 Gene Expression on T-Cells and Natural Killer Cells Declines in Conjunction with a Reduction in PD-1 Protein during the Intensive Phase of Tuberculosis Treatment. PLOS ONE. 2015;10:e0137646.

3. He S, Wang F, Yang L, Guo C, Wan R, Ke A, et al. Expression of DNMT1 and DNMT3a Are Regulated by GLI1 in Human Pancreatic Cancer. PLOS ONE. 2011;6:e27684.

4. Wang X, Spandidos A, Wang H, Seed B. PrimerBank: a PCR primer database for quantitative gene expression analysis, 2012 update. Nucleic Acids Res. 2012;40:D1144–9.

5. Amani Abkenari S, Safdarian L, Amidi F, Hosseini A, Aryanpour R, Salahi E, et al. Metformin improves epigenetic modification involved in oocyte growth and embryo development in polycystic ovary syndrome mice model. Mol Reprod Dev. 2021;88:817–29.
